# Supplementary material for: Identification of photoperiod-induced specific miRNAs in the adrenal glands of Sunite sheep (Ovis aries)
Source: Front Vet Sci. 2022 Jul 22;9:888207. doi: 10.3389/fvets.2022.888207 (PMC9354845; doi:10.3389/fvets.2022.888207)
Supplement: Supplementary file 1 [file Data_Sheet_1.ZIP › Supplementary file/miRDeep2 file.zip!/miRDeep2 file/Know_Structure/LP42A2/oar-mir-150.pdf]

miRBase precursor	: oar-mir-150
Total read count	: 741
oar-miR-150 read count	: 741
remaining reads	: 0

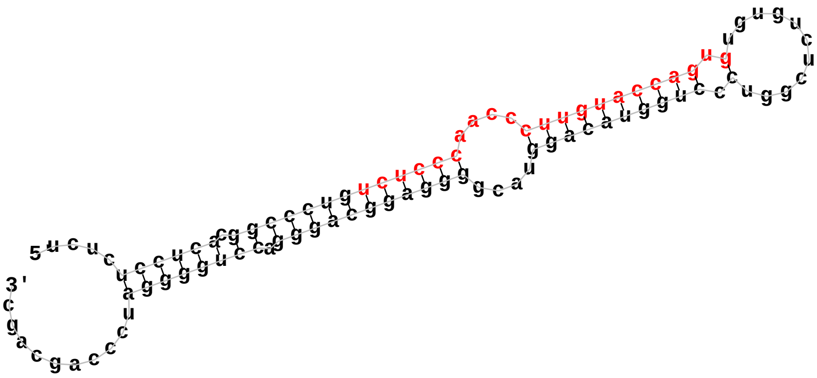

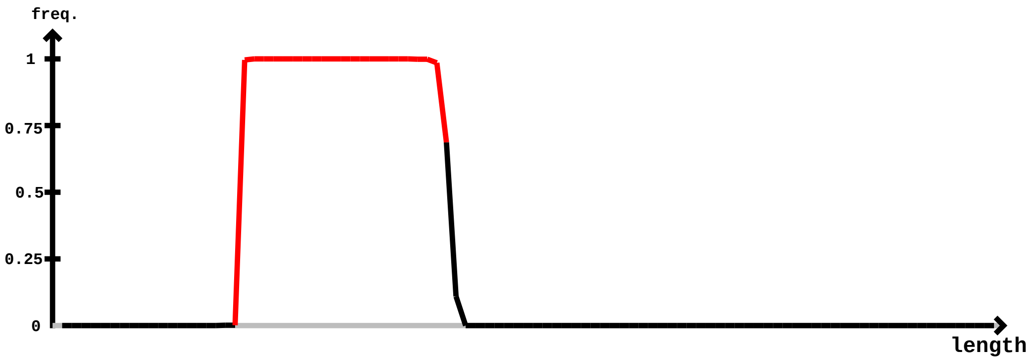


```
                  oar-miR-150
5'- ucucuccucacggcccugucucccaacccuuguaccagugugugucucggucccugguacagguacggggaggcagggaccuggggaucccagcagc   -3'    exp
    ....((((((.(((((((((((((....((((((((((.(...........).))))))))))....))))))))))).))))))))..........    reads      mm          sample
    ................ugucucccaacccuuguaccag...........................................................    1          0           seq
    ..................ucucccaacccuuguacc.............................................................    1          0           seq
    ..................ucucccaacccuuguaccag...........................................................    9          0           seq
    ..................ucucccaacccuuguaccagu..........................................................    219        0           seq
    ..................ucucccaacccuuguaccagug.........................................................    427        0           seq
    ..................ucucccaacccuuguaccagugu.......................................................    81         0           seq
    ...................cucccaacccuuguaccagu..........................................................    2          0           seq
    ...................cucccaacccuuguaccagug.........................................................    1          0           seq
```
